# Smartphone addiction and its associated factors among freshmen medical students in China: a cross-sectional study

**DOI:** 10.1186/s12888-022-03957-5

**Published:** 2022-05-02

**Authors:** Huan Liu, Zhiqing Zhou, Ergang Zhu, Long Huang, Ming Zhang

**Affiliations:** 1 Department of Hemodialysis, The First Affiliated Hospital of Wannan Medical College (Yijishan Hospital of Wannan Medical College, Wuhu, 241001 Anhui China; 2 Department of Nursing, The First Affiliated Hospital of Wannan Medical College (Yijishan Hospital of Wannan Medical College, Wuhu, 241001 Anhui China; 3 School of Comprehensive Foundation, Wannan Medical College, Wuhu, 241002 Anhui China; 4 School of Humanities and Management, Wannan Medical College, Wuhu, 241002 Anhui China; 5 School of Innovation and Entrepreneurship, Wannan Medical College, Wuhu, 241002 Anhui China

**Keywords:** Smartphone addiction, Freshmen medical students, China

## Abstract

**Background:**

With smartphone use widespread worldwide, smartphone addiction is an emerging epidemic. This study aims to investigate the prevalence of smartphone addiction among freshmen medical students and to explore its association with personal factors, mental health, and professional identity.

**Methods:**

This cross-sectional study was conducted from October 10th to November 10th, 2020 and included 2,182 first-year college students at Wannan Medical College, China. The smartphone addiction test, professional identity, and a 12-item general health questionnaire were used for this cross-sectional survey. Pearson’s correlation coefficient (r) was employed to examine the correlations between smartphone addiction and mental health and professional identity. Binary logistic regression analysis was carried out to assess the factors influencing smartphone addiction. Of the 2,182 students, 866 (39.7%) were identified as having smartphone addiction. The logistic regression analysis shows that four factors (professional identity scale, poor mental health, smartphone use before sleep, and perceived study pressure) were significantly associated with smartphone addiction.

**Conclusions:**

This cross-sectional study suggests that smartphone addiction is common among Chinese freshmen medical students. Smartphone addiction was common among the freshmen medical students surveyed. The findings imply that promotional programs, aimed at enhancing mental health and professional identity among freshmen medical students, help to reduce smartphone addiction in this population.

## Introduction

In the last decade, the number of smartphone users has grown exponentially and has become an indispensable part of our daily lives. According to the 45^th^ Statistical Report on China’s Internet Development, the current total number of Chinese Internet users is 904 million, and nearly 99.3% of them use their smartphones to connect to the Internet as of March 2020 [1]. Innovations in smartphone functions—including gaming apps, social media apps, online banking, compact digital cameras, social networks, global positioning system (GPS) navigation, portable media players, and shopping—have been a factor in the frequent use of smartphones. Although the widespread use of smartphones has brought many conveniences to our lives, the problems caused by their excessive use cannot be ignored.

Smartphone addiction, also referred to as “pathological smartphone use” or “smartphone dependence,” is defined as an people’s uncontrollable use of their smartphones; it can lead to serious harmful activity at work, while studying, and in daily life. Smartphone addiction has the properties of saliency, impulsivity, and withdrawal symptoms [2]. It is widely acknowledged that smartphone addiction not only adversely affects mental health, but can also be detrimental to learning, life, and physical health. Further, smartphone addiction has been associated with many health outcomes including fatigue [3], headaches[4], musculoskeletal pain[5], blurred vision [6], and poor sleep quality [7]. In addition, smartphone addiction can seriously affect college students’ academic performance [7], interpersonal competence [8], and emotional problems [9]. Smartphone addiction has become a serious public health concern that urgently requires immediate prevention and intervention. Although extensive research has been conducted on smartphone addiction in the past few years, most studies have focused primarily on college students [10–12].

Medical students face longer learning durations, higher academic strain, and greater clinical practice pressure than students in other disciplines, all of which have a negative impact on their psychological well-being and, most likely, lead to smartphone addiction [13]. Thus, it is important to pay close attention to smartphone addiction in Chinese medical students. In contrast, few studies in China have investigated the prevalence of smartphone addiction among medical students. In a cross-sectional study by Chen et al. (2017), the prevalence of smartphone addiction in Chinese medical students was 29.8% [14]. Smartphone addiction was found among 36.8% of newly admitted undergraduate medical students in Nepal [15]. In the present study, we aim to evaluate the status of smartphone addiction among freshmen medical students in China and to explore the relationship between smartphone addiction, mental health, and professional identity.

Past studies have revealed some components of smartphone addiction [16, 17]. However, the evidence of smartphone addiction and mental health among freshmen in Chinese medical schools is limited. Medical colleges are where medical students are likely to experience professional medicine for the first time, and college is an important transitional period for student growth. Due to the expansion of the Chinese education system, the employment rate of medical graduates has become significantly lower than before. Medical students in China experience substantial stress, which leads to numerous challenges, such as mental health problems [18]. However, the evidence between smartphone addiction and mental health among freshmen in Chinese medical schools is limited.

Professional identity among medical students refers to their acceptance and recognition of their chosen major and their willingness to devote themselves to learning with a positive attitude and positive behavior [19]. Medical college is where medical students are likely to experience professional medicine for the first time. Their professional identity is very important because it will influence their school learning and clinical practice. Wang et al. (2019) found a significant, positive correlation between professional identity and mental health [20] in Chinese medical students. We hypothesize that professional identity is one of the influencing factors of smartphone addiction in medical students.

A high prevalence of smartphone addiction among medical students is worrisome because it may affect student behavior, cause learning burnout, and ultimately affect academic performance and graduation. Therefore, the smartphone addiction of medical students is not only related to physical and mental health, but also has an a critical impact on the doctor–patient relationship and the quality of medical care they will provide in the future. A few studies on the influencing factors of smartphone addiction have found that gender [14], age [21], monthly income from one’s family [10], negative emotions [22], high impulsivity, and narcissism [23] could facilitate smartphone addiction. The current literature indicates that smartphone addiction prevention should pay attention to early detection of risk factors and intervention for addicted students; smartphone addiction in Chinese freshman medical students has been far less studied. Considering the seriousness of smartphone addiction and the rising incidence, research on smartphone addiction in freshman medical students is limited, and the relationship between smartphone addiction status and influencing factors needs to be probed more in depth. Hence, we aimed to scrutinize the prevalence of smartphone addiction in Chinese freshman medical students and to explore its association with demographic variables, mental health, and professional identity to provide a reference to reduce the prevalence of smartphone addiction.

## Materials and methods

### Participants

We used convenience sampling to gather the data. This is a network-based, cross-sectional study on first-year college students conducted in Fall 2020 at a medical school in Anhui Province. The medical students participated voluntarily and anonymously. We collected a total of 2,222 questionnaires and excluded 40 due to poor quality. Finally, we retained 2,182 valid questionnaires, which corresponded to an overall effective response rate of 98.20%, of which 1,009 (46.2%) were males and 1,173 (53.8%) were females. The age of the participants varied from 17 to 22, with a mean age of 19.16 (SD = 1.21).

### Measurement

#### Demographic characteristics

Based on the relevant literature review, the form included questions on one’s gender, age, college, being an only child or having siblings, place of residence, being a student leader (or not), and having fallen in love (or not).

#### Smartphone addiction

We measured the degree of smartphone addiction using the Smartphone Addiction Scale-Short Version (SAS-SV), which is a validated scale developed by Kwon in 2013 [24]. It is a self-report scale consisting of 10 items, and each item is rated on a 6-point Likert scale ranging from 1 to 6 (1 = strongly disagree; 6 = strongly agree). The total score is derived by adding the 10 items together. The overall SAS-SV score ranges from 10 to 60, and a higher SAS-SV score indicates a greater level of smartphone addiction. In this study, we adopted a cutoff point of 31 for males and 33 for females to classify smartphone addiction. The Cronbach’s alpha in the present study was 0.81.

#### Professional identity

We used the Chinese version of professional identity, translated and revised by Qin Panbo [25]; it includes four dimensions: cognition, emotionality, behavior, and fitness. The questionnaire consists of 23 items with scores ranging from 23 to 115 based on a 5-point Likert scale (5 = “complete conformity,” 4 = “conformity,” 3 = “neutral,” 2 = “inconformity,” and 1 = “complete inconformity”). A higher PIQUS score indicates a greater level of professional identity. In this study, the Cronbach’s alpha was 0.898.

#### The 12-item general health questionnaire (GHQ)

We used the GHQ-12, which has 12 items, to assess the medical students’ mental health [26]. The GHQ-12 is a self-report questionnaire that indicates how often the participants have felt a certain way in the past 2 weeks. All items are answered on a 4-point Likert scale ranging from 0 (not at all and no more than usual) to 1 (rather more than usual and much more than usual), and the final score ranges from 0 to 12. The results imply that the higher the score, the more mental health problems a student has. A GHQ-12 total score ≥ 3 signals poor mental health. In this study, the Cronbach’s alpha was 0.828.

### Data collection

We collected the data through an online survey from October 10th to November 10^th^, 2020. To facilitate the completion of the questionnaires, the questionnaire was answered through an online survey platform, “Questionnaire Star,” and counselors and teachers directly sent links to the students. All participating medical students needed to use “Questionnaire Star” to submit the questionnaire, and the same IP could only be submitted once. Before issuing the questionnaire, the electronic informed consent form for the medical students was obtained. The students were told that their participation was entirely voluntary and they were guaranteed confidentiality, and they could withdraw from the study at any time without a reason. The average time needed to complete the questionnaire was approximately 15 to 25 min. We carried out a preliminary pilot experiment with 30 freshmen medical students in Wannan Medical College, and the Cronbach’s alpha coefficient of the questionnaire’s internal consistency reliability was 0.88, indicating that the questionnaire can accurately measure the degree of smartphone addiction of freshmen medical students.

### Statistical analysis

In this study, we used “Questionnaire Star” to directly export data in a Microsoft Excel format, which we then exported into SPSS 20.0 software for statistical analysis. Continuous variables are represented as the means ± standard deviations, and categorical variables are portrayed as frequencies and percentages. We performed the χ2-test to compare differences in characteristics between the smartphone addiction group and the normal groups. We employed Pearson’s correlation coefficient (r) to explore the relationship between smartphone addiction and professional identity. We used forward stepwise binary logistic regression analysis to determine the predictors of smartphone addiction and to calculate the odds ratio (OR) and 95% confidence intervals (CIs). We considered a two-tailed P value of less than 0.05 to be statistically significant.

### Ethics

This study was reviewed and approved by the Ethics Committee of Wannan Medical College, China. We conducted the research protocol and procedures following the guidelines and principles of the Declaration of Helsinki (as revised in 2013). We obtained electronic informed written consent from all participants prior to filling out the questionnaire.

## Results

### The sociodemographic characteristics of the participants

We investigated a total of 2,182 college students in this study, of which 1,009 (46.2%) were males and 1,173 (53.8%) were females. Their ages ranged from 17 to 22, with a mean of 19.65 ± 0.83. Of the participants, 57.1% lived in rural areas, 27.5% lived in towns, and 15.3% lived in cities. Of the participants, 38.1% perceived high study pressure, 52.0% perceived medium study pressure, and 9.9% perceived low study pressure. In addition, 45.1% had high satisfaction with the profession, 50.3% had medium satisfaction with the profession, and 4.7% had medium satisfaction with the profession. The demographic characteristics of the participants (i.e., sex and residence) are described in Table 1.

**Table 1:** Univariate analysis of the participants’ demographic and mental health characteristics (*N* = 2,182)

Variables	Smartphone addiction (%)			χ^2^	*P*
	**No**	**YES**	**Total**		
	1316(60.3)	866 **(**39.7**)**	2182 **(**100.0**)**		
**Gender**				1.011	0.315
Male	620 (61.4)	389 (38.6)	1009 (46.2)		
Female	696 (59.3)	477 (40.7)	1173 (53.8)		
**Place of residence**				6.096	0.047
Rural area	728 (58.4)	519 (41.6)	1247 (57.1)		
Town	369 (61.4)	232 (38.6)	601 (27.5)		
City	219 (65.6)	115 (34.4)	334 (15.3)		
**Perceived study pressure**				49.437	< 0.0001
Low	154 (71.6)	61 (28.4)	215 (9.9)		
Medium	736 (64.8)	400 (35.2)	1136 (52.0)		
High	426 (51.3)	51.3 (48.7)	831 (38.1)		
**Satisfaction with profession**				26.251	< 0.0001
Low satisfied	55 (53.9)	47 (46.1)	102 (4.7)		
Medium satisfied	610 (55.6)	487 (44.4)	1097 (50.3)		
Highly satisfied	651 (66.2)	332 (33.8)	983 (45.1)		
**Smartphone use before sleep**				49.115	< 0.0001
No	176 (82.6)	37 (17.4)	213 (9.8)		
Yes	1140 (57.9)	829 (42.1)	1969 (90.2)		
**Student leader**				7.047	0.008
No	903 (58.5)	640 (41.5)	1543 (70.7)		
Yes	413 (64.6)	226 (35.4)	639 (29.3)		
**Have fallen in love**				0.245	0.621
No	997 (60.6)	648 (39.4)	1645 (75.4)		
Yes	319 (59.4)	218 (40.6)	537 (24.6)		
**Psychological problems**				43.89	< 0.0001
No	934 (56.4)	722 (43.6)	526 (24.1)		
Yes	382 (72.6)	144 (27.4)	1656 (75.9)		

### Factors associated with smartphone addiction in the univariate analysis

We detected smartphone addiction in 39.7% (866/2,182) of the respondents based on the SAS-SV. The results of the χ^2^ test are presented in Table 1. The statistical analysis revealed no significant relationship between smartphone addiction and sociodemographic traits, such as gender or having fallen in love (or not) (all *p* > 0.05). However, there were significant differences in place of residence, perceived study pressure, professional satisfaction, smartphone use before sleep, whether or not students are leaders, and mental health (*P* < 0.001)**.**

### The relationship between smartphone addiction and professional identity of freshmen

There were statistically significant, positive correlations between the professional identity scale, cognition scale, emotionality scale, and fitness scores and smartphone addiction scores (r = 0.794, *p* < 0.001; r = 0.448, *p* < 0.001; r = 0.566, *p* < 0.001; r = 0.710, *p* < 0.001; and r = 0.640, *p* < 0.001, respectively) (Table 2).

**Table 2:** The correlations between smartphone addiction and professional identity

**Variables**	Smartphone addiction	
	r	*P value*
Cognition	0.448**	< 0.001
Emotionality	0.566**	< 0.001
Behavior	0.710**	< 0.001
Fitness	0.640**	< 0.001
Professional identity scale	0.794**	< 0.001

### Factors associated with smartphone addiction

The results point to a significant correlation between smartphone use before sleep (OR = 3.120, 95% CI = 2.147– 4.536), perceived high study pressure (OR = 2.005, 95% CI = 1.430–2.811), and smartphone addiction (Table 3).

**Table 3:** Logistic regression analysis for factors associated with smartphone addiction

Variable	*B*	*SE*	*Wald*	*P*	*OR*	*95% CI*
Professional Identity Scale	-0.019	0.004	26.830	0.000	0.982	0.975–0.988
Poor mental health	0.517	0.115	20.142	0.000	1.677	1.338–2.102
Smartphone use before sleep	1.138	0.191	35.551	0.000	3.120	2.147–4.536
Perceived study pressure			29.985	0.000		
Medium	0.235	0.169	1.925	0.165	1.265	0.908–1.762
High	0.696	0.172	16.287	0.000	2.005	1.430–2.811
Constant	-0.719	0.409	3.092	0.079	0.487	

We employed the GHQ-12 to assess freshmen medical students’ mental health. The total outcomes revealed a significantly positive Pearson’s correlation coefficient between smartphone addiction and poor mental health (OR = 1.677, 95% CI = 1.338–2.102). We measured professional identity based on the Professional Identity Scale, and the results suggest that professional identity is a protective factor for smartphone addiction (OR = 0.982, 95% CI = 0.975–0.988).

## Discussion

The prevalence of smartphone addiction among the freshmen medical students in this study was 39.7%, which is slightly higher than another study that found that 29.8% of medical students in China had smartphone addiction [14]. Interestingly, in our study, the correlation analysis showed that perceived study pressure was positively correlated with smartphone addiction, whereas gender was not correlated with smartphone addiction. The changes in learning and lifestyle brought about by the COVID-19 pandemic have caused people to completely rely on the Internet and smart devices such as tablets, laptops, and mobile phones. This total dependence has proven to be a form of addiction [27]. Recently, concern was expressed about the increase in smartphone use among students due to the decrease in, or absence of, supervision. In the context of the COVID‐19 pandemic, participants may attempt to use smartphones to reduce stress and alleviate poor mood [28], which can cause higher risks for increases in smartphone use and smartphone addiction.

Smartphones are the easiest available substances for managing stress and can lead to addiction. Crisis management procedures—including isolation; social distancing; continuous confinement; wearing masks; the cancellation of family, social and cultural activities—and individual health issues are all potential stressors [29, 30]. The negative impact of COVID-19 on mental health extends beyond health-related fears [31] and can lead to negative psychological effects [32, 33]. The COVID-19 pandemic and the series of effects it has engendered such as social distancing, lockdowns, and home confinement [34] have caused many changes in our mental and behavioral health. Hence, COVID-19 could strengthen the use of addictive substances as strategies to cope with or relieve stress [35, 36]. Nevertheless, the findings of high prevalence indicate that smartphone addiction may be a serious problem that has a significant impact on medical students’ own lives and studies. The current COVID-19 crisis requires new ways to identify health conditions while maintaining the principles of reducing human contact and pollution. In this case, a proactive approach to monitoring smartphone use in the student is essential.

This study implies that professional identity is a positive personal trait and an important protective factor. It can prevent individuals from becoming affected by perceived stress, thereby reducing their negative emotions and the possibility of developing smartphone addiction. Many studies have found that the professional identity of medical students has a positive effect on their learning motivation, academic success, and professional development; it is also the most vital factor influencing their future career choices and professional achievements [37, 38]. Professional identity refers to students’ acceptance and recognition of their major, as well as their willingness to learn and explore with a positive attitude and positive behavior. Medical students are the reserve force of medical developments in the future, and their professional identity is crucial for the quality training of modern medical talent. The professional identity of nursing students can improve their self‐confidence and enhance their professional skills [39]. Professional identity can help individuals to deal with stress effectively and to achieve better adaptation and development. When faced with stress, medical college students with greater levels of professional identity might recover quickly with positive psychological capacity, make greater efforts in the pursuit of success, and have positive expectations and attributes for the outcomes. Thus, improving their professional identity can play a role in preventing smartphone addiction.

Poor mental health is positively correlated with smartphone addiction [40, 41]. Poor mental health is one of the most crucial risks leading to smartphone addiction. There is a significant correlation between perceived vulnerability to disease (PVD) and emotional distress [42]. The fear of contagion is one of the causes of anxiety, depression, and stress in the general population. University students’ response to the pandemic deserves further attention. The sudden changes in “university” habits (i.e., poor interactions with teachers and colleagues, difficulty adapting to online learning), the loss of social networks, and other issues have seriously affected the physical and mental health of university students. A study carried out in China found that approximately one-third of the respondents had moderate-to-severe anxiety, a high prevalence of sleep disorders, and widespread symptoms of anxiety disorder, especially in young people and health professionals [43, 44]. Due to the portability and flexible choices of smartphone applications, individuals with poor mental health will make up for the shortcomings in their real social lives by using various smartphone applications. Elhai et al. found that COVID-19 anxiety was related to the severity of depression, anxiety, and problematic smartphone use (PSU) [45]. It is increasingly becoming clear that excessive use of smartphones has a negative impact on health.

In line with previous studies [46–48], we found that perceived stress was positively correlated with smartphone addiction among medical freshmen students. The impact of perceived stress on smartphone addiction has begun to gain support in the research world [46, 49]. As a special group of college students, medical students experience a high level of stress that could be due to academic burden, the frequency of exams, and a long academic curriculum [50]. Addictive behavior may be a way to reduce strain or relieve negative emotions. Individuals who feel more stress are more likely to develop smartphone addiction [48]. When faced with stress, medical students are more likely to use smartphones to mitigate stress [49]. Perceived stress could make individuals believe they are in a state of stress, which is considered to be a risk factor for the occurrence and recurrence of many addictions, such as drug abuse [51] and Internet addiction [52]. The results show that it is very important for counselors to improve medical college students’ professional identity in the context of smartphone addiction. In addition, for future clinical practitioners, we must pay attention not only to the symptoms of smartphone addicts themselves, but also to their stress and mental health.

Prolonged contact with smartphone screens reduces sleep time and sleep efficiency. The COVID-19 pandemic home quarantine brought about a sudden and different lifestyle, with many students watching movies/series most of the time. The overuse of smartphones usually results in smartphone addiction.

Some limitations must be admitted. First, we conducted this study through an online survey; therefore, it is possible that some data could be erroneous. Second, this study was cross-sectional and hence we cannot infer causality. Meanwhile, the representativeness of the sample may limit the general validity of our findings because our participants were from the same university. Third, we gathered the data during the COVID-19 pandemic, so it is difficult to separate the effect from the given sample. Further longitudinal follow-up studies should be conducted to explore smartphone addiction status and its associated factors among medical students in China.

In sum, high smartphone addiction scores were significantly correlated with low professional identity, poor mental health, smartphone use before sleep, and perceived study pressure. Therefore, it is necessary to perform a large-scale longitudinal study to further establish the causal relationship between smartphone addiction, mental health, and professional identity.

## Data Availability

The datasets used and/or analyzed during the current study are available from the corresponding author (H.L. or M.Z.) upon reasonable request.
